# Antiangiogenic therapy using endostatin increases the number of ALDH+ lung cancer stem cells by generating intratumor hypoxia

**DOI:** 10.1038/srep34239

**Published:** 2016-10-05

**Authors:** Yang Yu, Yu-yi Wang, Yi-qin Wang, Xia Wang, Yan-Yang Liu, Jian-Tao Wang, Chi Du, Li Wang, Mei Li, Feng Luo, Ming Jiang

**Affiliations:** 1 Department of Medical Oncology, Cancer Center and State Key Laboratory of Biotherapy, West China Hospital of Sichuan University, Chengdu, Sichuan Province, China; 2 Department of Medical Oncology, Ganzhou City People’s Hospital, Ganzhou, Jiangxi Province, China; 3 Department of Radiation Oncology, The Second People’s Hospital of Neijiang, Neijiang, Sichuan Province, China

## Abstract

Antiangiogenic therapy is becoming a promising option for cancer treatment. However, many investigations have recently indicated that these therapies may have limited efficacy, and the cancers in most patients eventually develop resistance to these therapies. There is considerable recently acquired evidence for an association of such resistance with cancer stem-like cells (CSLCs). Here, we used xenograft tumor murine models to further suggest that antiangiogenic agents actually increase the invasive and metastatic properties of lung cancer cells. In our experiments with murine lung cancer xenografts, we found that the antiangiogenic agent endostatin increased the population of ALDH+ cells, and did so by generating intratumoral hypoxia in the xenografts. We further showed endostatin to cause an increase in the CSLC population by accelerating the generation of tumor hypoxia and by recruiting TAMs, MDSCs and Treg cells, which are inflammatory and immunosuppressive cells and which can secrete cytokines and growth factors such as IL-6, EGF, and TGF-β into the tumor microenvironment. All these factors are related with increased CSLC population in tumors. These results imply that improving the clinical efficacy of antiangiogenic treatments will require the concurrent use of CSLC-targeting agents.

Since angiogenesis plays an important role in the growth and metastasis of solid tumors^1^, antiangiogenic therapy is becoming a promising option for cancer treatment. However, many observations indicate that this type of therapy may have limited efficacy, and in most patients the cancers eventually display resistance to this treatment^2^,^3^. Recent studies have suggested that although the agents used in such therapy typically inhibit primary tumor growth, lasting responses are rare, with only a moderate increase in progression-free survival and little benefit in overall survival^2^. In addition, when antiangiogenic agents are administered on an intermittent schedule, tumor regrowth is sometimes seen during drug-free periods^4^ or upon discontinuation of the treatment^5^.

There is considerable recently acquired evidence for the association of resistance to antiangiogenic therapy with complex changes in the tumor microenvironment, in which precise and complex “cross-talk” occurs between the tumor cell and other components of the tumor microenvironment^6^. On the one hand, administration of antiangiogenic agents has been shown to accelerate intratumoral hypoxia, and hypoxia has been shown to regulate the transcriptional activity of hypoxia-inducible factors 1 (HIF-1α). Moreover, HIF-1α has been shown to modulate each step of the metastatic process^7^ and to play an important role in the stimulation of cancer stem-like cells (CSLCs) or cancer stem cells (CSCs)^8^,^9^,^10^,^11^. On the other hand, the hypoxic microenvironment in tumors attracts inflammatory cells and immunosuppressive cells such as tumor-associated macrophages (TAMs)^12^, myeloid-derived suppressor cells (MDSCs)^13^ and regulatory T cells (Treg cells)^14^, and those cells through paracrine many cytokines factors, such as IL-6, IL10, EGF, SCF, TNF-α and TGF-β, which can increase and enrich CSLCs through continuous activation of pluripotent and self-renewal pathways such as the Hedgehog, Notch and Wnt/β-catenin pathways^15^,^16^,^17^,^18^.

A large number of studies have shown that many tumors are maintained by a subpopulation of cells, in particular CSLCs or CSCs, which play a pivotal role in tumor initiation, recurrence and metastasis, and hence constitute one of the primary causes for resistance to antiangiogenic agents^19^. Although certain tumor cells escape from the hostile hypoxic environment, others become more hypoxia tolerant^20^,^21^,^22^,^23^. Notably, CSLCs home in on hypoxic regions of tumors, where they can sustain self-renewal potential^24^. However, other studies have documented that CSLCs are also present in perivascular niches, release angiogenic factors in hypoxic conditions, and establish a permissive vascular niche^20^,^25^.

Lung cancer cells expressing various molecules such as CD133, CD166, aldehyde dehydrogenase (ALDH), CXCR4, and GLDC have been shown to demonstrate phenotypic characteristics of CSLCs^26^,^27^,^28^,^29^. However, identification of human lung CSLCs has been hampered by the lack of reliable normal lung epithelial stem cell markers^30^. ALDH enzymes constitute a family of intracellular enzymes that participate in cellular detoxification, differentiation, and drug resistance through the oxidation of cellular aldehydes^31^, and research has shown that CSLCs is enriched in ALDH+ cells^32^. Furthermore, expression and activity of ALDH has been found in stem cells of many tumor types such as colon cancer, renal cancer, malignant melanoma, and breast cancer^33^,^34^,^35^,^36^. ALDH has recently been expressed in murine embryonic lungs and has been reported to select for human lung CSLCs^29^,^37^,^38^,^39^. Moreover, being ALDH+ has been shown to contribute to the invasion, migration, tumorigenicity and drug-resistance capacities of two human Non Small Cell Lung Cancer(NSCLC) cell lines^39^. Together, these findings suggest that ALDH proteins levels or enzymatic activity may serve as candidate markers for lung CSLCs.

Endostatin, a broad-spectrum angiogenesis inhibitor, works in various ways including by inhibiting the binding of VEGF to endothelial cells, the migration of endothelial cells, and the Wnt pathway^40^,^41^,^42^. However, our studies showed that the effects of monotherapy with endostatin on tumor growth were transient and there was no significant benefit for survival of tumor-bearing mice^43^,^44^. In our previous study, the administration of endostatin was found to facilitate intratumoral hypoxia, promoting recruitment of TAMs, MDSCs and Treg cells, which can secrete many cytokines^45^. The relationship between the endostatin-induced increase of the number of CSLCs in tumors and antiangiogenic therapy resistance remains to be determined.

In this study, we first established xenograft tumor murine models, and then treated the tumors with endostatin. We demonstrated that endostatin increases the number of CSLCs, which was related with generated intratumoral hypoxia and attracted inflammatory cells and immunosuppressive cells.

## Materials and Methods

### Cell line and culture conditions

mice Lewis lung carcinoma (LLC) cells were obtained from Sichuan University (Chengdu, Sichuan, China) and were maintained in RPMI 1640 (Gibco) supplemented with 10% fetal bovine serum (Gibco) and 1% penicillin-streptomycin (Gibco). The cells were incubated in a humidified incubator supplied with a 5% CO2 atmosphere at 37 °C.

For the hypoxia assay, LLC cells were stimulated with CoCl2 (100 μM; Sigma) for 24–48 h, and, after each time point, were assayed for ALDH activity by using the ALDEFLUOR assay.

### Tumor models and treatment protocol

All mice experiments were conducted in accordance with standard operating procedures approved by the Animal Ethics Committee of Sichuan University. Wild-type C57BL/6 mice at 5–7 weeks of age were purchased from Huafukang Bio-Technology (Beijing, China). The mice were subcutaneously injected with 3 × 10^5^ LLC cells in the right hind flank^46^. When tumors reached an average volume > 5 mm^3^, the mice were randomly distributed into two groups (n = 15/ group): the control group received normal saline (NS), and the endostatin group received 20 mg/kg endostatin^47^ (recombinant human endostatin, Simcere Medgenn Bio-Pharmaceutical Company, Yantai, Shan-dong Province, China). Normal saline or endostatin was administered daily by intraperitoneal (i.p.) injection, and the mice were monitored every 3 days for tumor development. The volume was calculated using the formula V = a × b2/247, where a and b are the long and the short tumor diameters, respectively^1^. Three mice from each group were sacrificed on day 8 after treatment, as well as on day 16 and on day 24 after treatment, to harvest tumor tissues for further study.

### Cell viability assay

Cell viability was assessed using an MTT assay. The cells (7 × 10 3 LLC cells) were seeded in 96-well flat-bottomed microtiter plates in six cultures. After an overnight incubation, the cells were treated with endostatin for 48 hours at 37 °C. MTT (Sigma) was prepared at 5 g/mL in phosphate-buffered saline (PBS) and added to each well, and the cells were then cultured for another 4 h at 37 °C. After removing supernatant, DMSO (Sigma) was added to each well, and the OD was measured at a wavelength of 570 nm using a Benchmark microplate reader (Benchmark Electronics, Angleton, TX, USA). The viabilities of the cells given the indicated treatments were compared to that of cells treated with the control.

### Immunohistochemistry

Formalin-fixed, paraffin-embedded tumor tissues were sectioned into adjacent 3 um layers, deparaffinized, rehydrated, incubated in 3% H2O2, and blocked with 5% bovine serum albumin. The sections were then incubated with a hypoxia-inducible factor 1-α (HIF-1α) antibody (diluted 1:100, mice monoclonal; CST, USA) and CD31 (diluted 1:100, mice monoclonal; CST, USA) overnight at 4 °C. After washing in phosphate buffered saline, the secondary antibody, biotinylated polyclonal rabbit anti-mice antibody (diluted 1:200; Dako, China), was applied. The DAB substrate kit (Dako, China) was used for the subsequent steps. Photographs were acquired by using a microscope (Leica, Wetzlar, Hessen, Germany) at ×200 magnification.For HIF-1α and CD31counting, at least five randomly selected regions for slides were analyzed^39^.

### ELISA

Tumor samples were collected for the assays from all mice on days 8, 16 and 24 after treatment. Total protein levels were quantified by a BCA assay (Thermo Scientific, Rockford, IL). Levels of VEGF (eBioscience, USA), EGF (R&D, USA), IL-6 (eBioscience, USA) and TGF-β (eBioscience, USA) were assessed by using commercially available mice ELISA kits according to the manufacturer’s instructions. A Benchmark microplate reader (Benchmark Electronics, Angleton, TX, USA) was used to read the colorimetric reaction.

### ALDEFLUOR assay and cell sorting

Single-cell suspensions were obtained by carrying out collagenase I (Sigma) dissociation of tumor tissues, via incubation with 1 mg/ml collagenase I for 1 hour at 37 °C. The ALDEFLUOR kit (01700; Stem Cell Technologies, NC, USA) was used for the immunofluorescent detection of intracellular ALDH enzyme activity. The ALDEFLUOR assay was carried out according to the manufacturer’s (Stem Cell Technologies) instructions. Briefly, cells were suspended in the ALDEFLUOR assay buffer containing the ALDH substrate BoDipy-aminoacetaldehyde (BAAA), and incubated for 40 min at 37 °C. To distinguish between ALDH+ and ALDH− cells, a fraction of the cells was incubated with the ALDH inhibitor diethylaminobenzaldehyde (DEAB). The cells were analysed and sorted with a Beckman MoFlo XDP cell sorter Beckman Coulter, Fullerton, CA, USA).

### Propidium iodide assay

Cell viability was evaluated using propidium iodide (PI) staining followed by flow cytometry. Single-cell suspensions were obtained by carrying out collagenase I (Sigma) dissociation of tumor tissues, via incubation with 1 mg/ml for 1 hour at 37 °C. Cells were washed twice with cold PBS and centrifuged at 300 × g for five minutes. The cells were then resuspended in binding buffer and incubated with 2 ug/ml PI (Sigma) for five minutes at room temperature. A total of at least 1 × 106 events were collected and analyzed by flow cytometry (BD).

### Sphere formation assay

For sphere formation, cells were grown in serum-free DMEM/F12 (1:1) (Invitrogen), supplemented with 1X-N2 supplement (Invitrogen), 10 ng/ml EGF (Peprotech) and 10 ng/ml bFGF (Peprotech); the cells were allowed to grow for fourteen days^48^. Cells were plated in ultra-low attachment 6-well plates (Corning, Lowell, MA, USA) at a density of 4000 cells per well. Images of the spheres were taken using a phase contrast microscope (Nikon) and the number of spheres > 50 mm in diameter were counted.

### *In vivo* tumor formation assay

Six-week-old wild-type C57BL/6 mice were used for the *in vivo* tumor formation assays. Indicated numbers of sorted ALDH+ or ALDH− cells from tumors were washed with serum-free DMEM-F12 and implanted subcutaneously in the right hind flank^49^.

### Reverse transcription PCR and quantitative real-time PCR

Expression of mRNA was analyzed by applying the quantitative RT-PCR (QPCR) (Takara) and SYBR green detection (Takara). Expression of GAPDH was used as the control. The primers used for the RT-PCR analysis are listed in Table 1.

### Flow cytometry analysis

Single-cell suspensions which were obtained from tumor tissues, were stained for surface markers using anti-mice CD11b-FITC (BD Biosciences, USA), Gr1-PE (BD Biosciences, USA), and F4/80-PE (MACS, Germany). The Treg cell assay was carried out according to the instructions of eBioscience, CA. The cells were first stained for surface markers using rat anti-mice CD4-FITC and CD25-APC. Next, intracellular staining was performed using rat anti-mice Foxp3-PE (eBioscience, CA). For intracellular cytokine staining, the cells were fixed and permeabilized with Cytofix/Cytoperm buffers (eBioscience, CA) for 60 min at 4 °C and then washed with permeabilization wash buffer (eBioscience, CA). Next, the cells were stained with rat anti-mice Foxp3-PE (eBioscience, CA). Finally, the cells were analyzed with a Beckman MoFlo XDP cell sorter and Submit software (Beckman Coulter, Fullerton, CA, USA).

### Statistical methods

To assess the statistical significance of differences between measured values, Student’s t-tests were performed. Survival curves for mice were calculated using the Kaplan-Meier method, and analysed using the log-rank test. All statistical analyses were performed with GraphPad Prism 5 software (GraphPad), SigmaPlot 12.3 software and SPSS19.0 statistical software. Data were presented as the mean ± standard deviation (SD). The data were considered statistically significant when the p value was less than 0.05.

## Results

### Anti-tumor efficacy of endostatin *in vivo*

**Figure 1:**
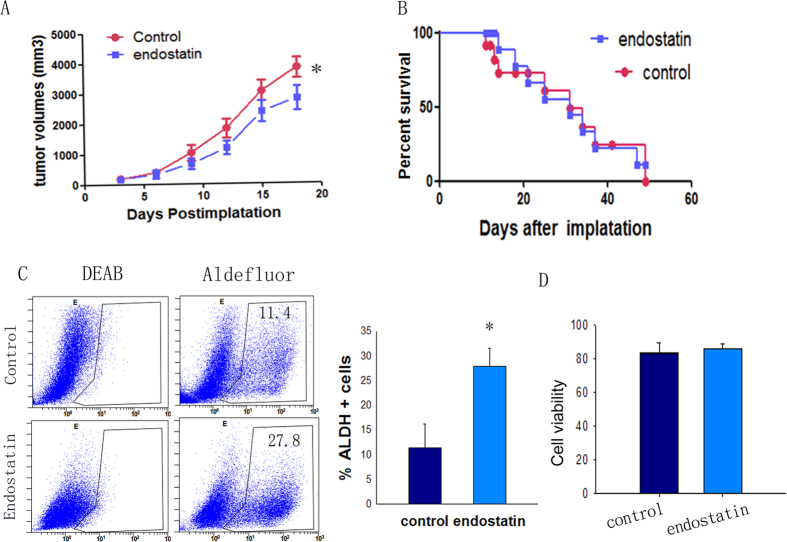
(**A**) Endostatin-treated tumors (blue) were significantly smaller than normal saline-treated tumors (red) at the end point (n = 11, p < 0.05). (**B**) Survival percent (n = 11, p > 0.05). (**C**) ALDH+ cell population (measured by using ALDEFLUOR assays) in the tumor was quantified by gating for ALDH+ cells (n = 5, *p < 0.05, **p < 0.01). (**D**) Cell viability was evaluated using propidium iodide (PI) staining followed by flow cytometry. n = 5, and data are shown as averages ± SD.

To determine whether antiangiogenic therapy with endostatin could inhibit tumor growth, we monitored the change of tumor volume. The treatment regimens were performed as described in Materials and Methods. As expected, a significant inhibition of tumor growth was observed in the endostatin treatment group compared with the control group (p < 0.05) (Fig. 1A). However, endostatin did not have a life-prolonging effect when comparing tumor-bearing mice between treatment and control groups (p > 0.05) (Fig. 1B). Moreover, all tumor-bearing mice died of tumor burden within 50 days of implantation.

### Endostatin treatment increases the population of ALDH+ cells in tumors

After propidium iodide (PI) staining, cell viability was determined by using flow cytometry. On average, the control group yielded an 83.30 ± 6.37% viability, whereas the treatment group yielded an 85.74 ± 3.04% viability. There was no significant difference between the viability of cells from the control group and that of the treatment group (p > 0.05) (Fig. 1D). Aldehyde dehydrogenase (ALDH) activity was assessed by using the ALDEFLUOR assay. The control and treatment groups had an average of 11.40 ± 4.81% and 27.80 ± 3.78% ALDH+ cells, respectively, Using DEAB (a specific ALDH inhibitor) provided a negative control (Fig. 1C). The percentage of ALDH+ cells in mice of the treatment group was significantly greater (p < 0.01) than that of the control group (Fig. 1C).

### ALDH+ cells isolated from tumor tissues exhibit characteristics of stem cells

**Figure 2:**
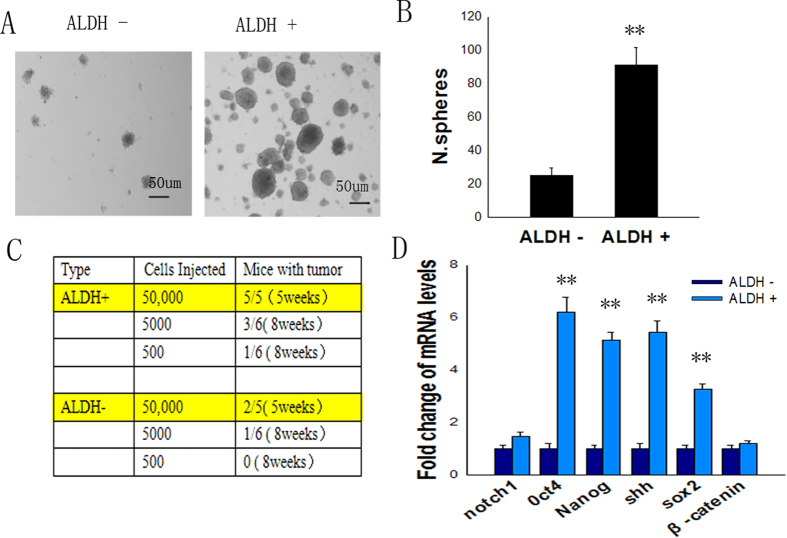
The ALDH+ LLC cell population displayed stem cell properties. FACS was used to separate LLC cells in tumor tissues into ALDH+ and ALDH− cell populations. (**A,B**) Spheroid formation assay. Cells kept in serum-free, low-adherence 6-well plates for 14 days (n = 6, p < 0.05). (**C**) Sorted ALDH+ and ALDH− cells were implanted into the flanks of mice. The number of tumors formed within the indicated time are tabulated (n = 5 or 6). (**D**) After the two freshly isolated populations were isolated, genes associated with stem cells were analyzed by qPCR. n = 3, and data are shown as averages ± SD. p < 0.05. *p < 0.05, **p < 0.01.

FACS was used to separate LLC cells in the tumor tissues into ALDH+ and ALDH− cell populations. To determine the tumorigenic potential of different subpopulations of cells, ALDH+ and ALDH− cells were separately harvested for further study. ALDH+ and ALDH− cells were subjected *in vitro* to sphere formation assays, and our findings indicated that ALDH+ cells formed spheres (91.67 ± 10.17) efficiently. In contrast, ALDH− cells formed spheres (25.00 ± 4.33) seldomly (p < 0.01) (Fig. 2B). In addition, the sphere formation assay revealed that ALDH+ cells were able to induce larger and more spheres (Fig. 2A,B). This result demonstrated that ALDH+ cells have a higher self-renewal capability than do ALDH− cells.

ALDH+ and ALDH− cells were subjected *in vivo* to tumorigenic experiments, and our findings indicated that subcutaneous injection of activated ALDH+ cells resulted in the development of more and larger tumors than did the injection of ALDH− cells (Fig. 2C). There was one successful tumor formation when 500 ALDH+ cells were injection to six of mice, whereas the same phenomenon could be observed only when a minimum of 5000 ALDH− cells were used in the same condition. In addition, tumors generated from the injection of ALDH− cells developed much more slowly than did those generated from the injection of ALDH+ cells. This result demonstrated that ALDH+ cells are more tumorigenic than are ALDH− cells.

We also compared the expression profiles of pluripotency genes and stem cell-related signaling pathway genes in ALDH+ cells to those in ALDH− cells using q-PCR. Our findings revealed that the ALDH+ population displayed a significantly higher expression of the pluripotency genes, such as Nanog (approximately 5.15-fold), OCT4 (approximately 6.20-fold), SOX2 (approximately 3.29-fold), and the stem cell-related signaling pathway gene sonic Hedgehog (Shh) (approximately 5.45-fold) (Fig. 2D). Together, these results indicated that the ALDH+ population showed expression profiles characteristic of CSLCs.

### Endostatin cannot increase the number of CSLCs *in vitro*

**Figure 3:**
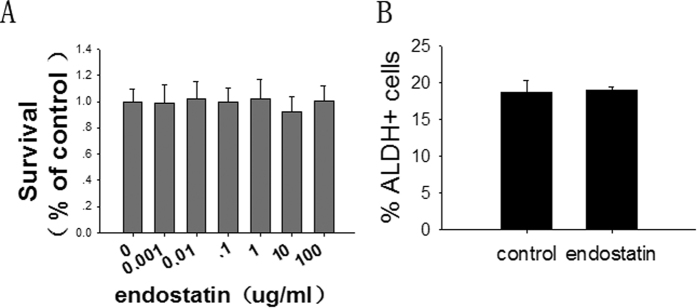
Endostatin does not increase the number of ALDH+ cells directly. (**A**) An MTT assay was used to measure cell viability in LLC cells following treatments with 0.001 to 100 μg/ml concentrations of endostatin (n = 6, p > 0.05). (**B**) LLC cells following treatment with 0.2 μg/ml endostatin. The population of ALDH+ cells (measured by ALDEFLUOR assays) in the tumor was analyzed. n = 3, and data are shown as averages ± SD, p > 0.05. *p < 0.05, **p < 0.01.

To gain insight into the mechanism by which endostatin regulates the number of lung CSLCs, we assessed the relationship between the Lewis lung carcinoma (LLC) cell line and endostatin by using the MTT and ALDEFLUOR assays. In the MTT assay, we found that there was no significant change in the viability of cells of the LLC cell line following treatments with 0.001 to 100 μg/ml concentrations of endostatin (p > 0.05) (Fig. 3A). We used the LLC cell line following endostatin,cocultured and then used the ALDEFLUOR assay. On average, 18.72 ± 1.53% of control cells and a statistically similar (p > 0.05) 19.00 ± 0.46% of endostatin-cocultured cells were found to be ALDH+ (Fig. 3B). This observation suggests that endostatin cannot directly increase the relative number of ALDH+ cells.

### Endostatin aggravates hypoxia in tumors

**Figure 4:**
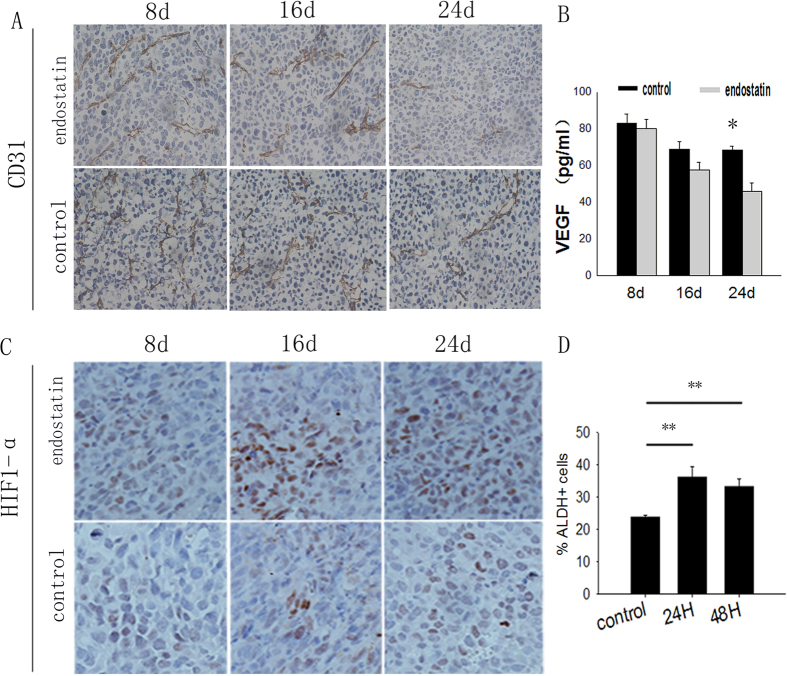
Endostatin treatment can induce hypoxia in tumors *in vivo*. (**A**)CD31 staining of blood vessels of tumors in 3 lung cancer tissues (×200). (**B**) Levels of human VEGF protein in NSCLC cells. mice VEGF protein in tumor tissue was assessed by using ELISA (n = 3, p > 0.05). (**C**) Tumors were detected by immunohistochemistry staining of HIF-1α in 3 lung cancer tissues (×200). *p < 0.05, **p < 0.01. (**D**) Cells were grown under normoxia or hypoxia for 1–2 d. n = 3, and data are shown as averages ± SD, p > 0.05.

To further confirm the mechanism by which endostatin regulates the population of lung CSLCs in mice, staining was carried out for the endothelial marker CD31. This staining revealed significantly fewer blood vessels in tumors from treated mice than in tumors from controls (Fig. 4A). Given the evidence that vascular endothelial growth factor (VEGF) may be involved^50^, we used an ELISA after each time point to analyze variations in the level of the VEGF in the tumor tissue of LLC tumor-bearing mice. We found that VEGF levels in tumor tissue decreased significantly on day 24 (p < 0.05) in the treatment group (45.72 ± 4.79) compared with controls (68.4 ± 2.14) (Fig. 4B).

To further study the changes in the tumor microenvironment hypoxia induced by antiangiogenic therapy, we examined the expression of HIF-1α in the tumor microenvironment in tumor tissue. On days 8, 16 and 24 after treatment, a higher expression of HIF-1α in the tumor microenvironment was found in the treatment group than in the control group (Fig. 4D).

We tested the effect of low levels of oxygen on lung ALDH+ cells *in vitro*. LLC cells were cultured under low oxygen levels and then assayed for ALDH activity by using the ALDEFLUOR assay. The percentage of ALDH+ cells in the tissue under low oxygen levels after 24 h (36.35 ± 3.113%) and 48 h (33.35 ± 2.17%) were significantly greater than the percentage of ALDH+ cells in normal oxygen levels (23.89 ± 0.45%) (P < 0.01). These findings support the hypothesis that antiangiogenic drugs stimulate the ALDH+ population by generating hypoxia in tumors.

### The percentages of TAM, MDSCs and Treg cells increase in LLC tumor-bearing mice

**Figure 5:**
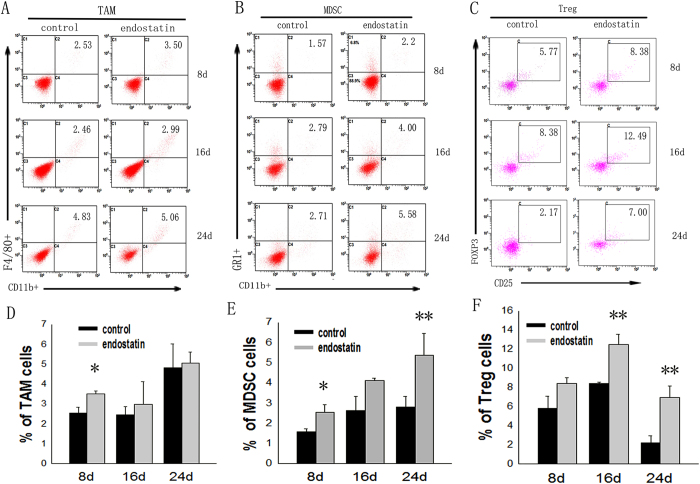
Quantification of TAMs, MDSCs and Treg cells in the primary tumor tissue. Single-cell suspensions were derived from tumor tissues, and each immune cell subset was analyzed by flow cytometry. (**A**) The levels of tumor-infiltrating TAM (F4/80+ CD11b+) cells were quantified by gating for F4/80+ CD11b+ cells. (**B**) The levels of tumor-infiltrating MDSCs (Gr1+ CD11b+) were quantified by gating for Gr1+ CD11b+ cells. (**C**) The levels of tumor-infiltrating Treg (CD4+ CD25+ Foxp3+) cells were quantified by gating for CD4+ CD25+ Foxp3+ cells. The levels of (**D**) TAMs, (**E**) MDSCs, and (**F**) Treg cells were comparable in the tumor-bearing mice with normal saline or endostatin. n = 3, and data are shown as averages ± SD, *p < 0.05, **p < 0.01.

FACS was used to assess the percentage of tumor-infiltrating tumor-associated macrophages (TAMs), myeloid-derived suppressor cells (MDSCs) and regulatory T cells (Treg cells) in tumor tissue. On day 8 of our experiments, there was a significant increase in the relative number of TAMs in the tumors of the group of mice treated with endostatin (3.50 ± 0.13%)compared with those of the control group (2.53 ± 0.29%) (p < 0.05) (Fig. 5A,D). Also, the percentage of MDSCs was significantly higher in the treated mice than in control mice on days 8 (2.53 ± 0.38 and 1.57 ± 0.16%) and 24 (5.37 ± 1.07 and 2.81 ± 0.51%) (p < 0.01) (Fig. 5B,E). Moreover, the percentages of tumor-infiltrating CD4 + CD25 + Foxp3 + Treg cells were also higher in the treated group than in the controls (Fig. 5C,F), with significant increases in the percentages of these Treg cells on days 16 (12.50 ± 1.07 and 8.38 ± 0.17%) and 24 (6.95 ± 1.17 and 2.17 ± 0.74%) (p < 0.01).

### Changes of the levels of cytokines related to inflammatory and immunosuppressive cells

**Figure 6:**
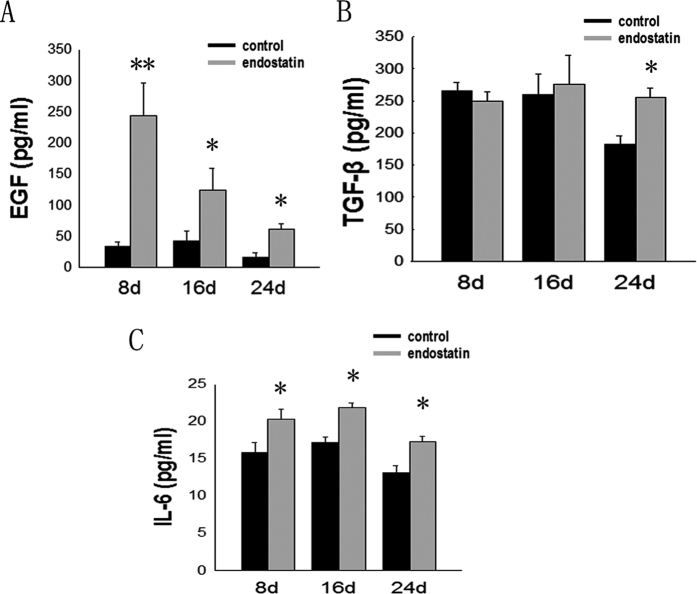
Changes in the levels of cytokines related to TAMs, MDSCs, and Treg cells in the tumor tissue of tumor-bearing mice. ELISA analyses for cytokines in tumor tissue samples of tumor-bearing mice from each group were carried out on days 8, 16 and 24 after treatment with endostatin. (**A**) EGF, (**B**) TGF-β, and (**C**) IL-6 levels in LLC tumor-bearing mice from each group at each time point. n = 3, *p < 0.05, **p < 0.01.

Given the evidence that many cytokines, such as EGF, IL-6 and TGF-β, are secreted by the above-mentioned immunosuppressive cells, such cytokines may be implicated in eliciting CSLCs and aggravating immunosuppression and inflammation of the microenvironment. We used ELISA to analyze changes in the levels of these cytokines in the tumor tissue of tumor-bearing treated and control mice. We found that EGF levels increased significantly in the treated group compared to the controls on day 8 (243.91 ± 51.64 and 33.38 ± 7.49), 16 (124.24 ± 34.60 and 41.72 ± 16.20) and 24 (61.39 ± 9.16 and 16.63 ± 6.67) (p < 0.05) (Fig. 6A). TGF-β levels were significantly different between the control groups and treated groups on day 24 (254.72 ± 14.77 and 182.34 ± 13.79) (Fig. 6B). IL-6 levels increased significantly in the treated groups compared with the control groups on day8 (20.30 ± 1.40 and 15.88 ± 1.26), 16 (21.87 ± 0.63 and 17.15 ± 0.77) and 24 (17.25 ± 0.77 and 13.12 ± 0.96) (p < 0.05) (Fig. 6C).

## Discussion

Many markers have been used to show that lung cancer cells demonstrate CSLC phenotypic characteristics. However, no single marker has been found to be universally applicable for human lung CSLCs. In this study, we applied a promising stem cell marker, ALDH activity, to identify and enrich for a subpopulation of lung cancer cells with many of the properties ascribed to CSLCs^8^. ALDH+ cells were shown in our study to have a higher self-renewal capability and tumorigenicity than ALDH− cells. Additionally, the ALDH+ population showed significantly higher expression levels of Nanog, OCT4, SOX2 and the sonic Hedgehog (Shh) gene. These results indicated that the expression and activity of aldehyde dehydrogenases (ALDHs) constitute a potential CSLC marker of lung cancer cells, and these results are consistent with the findings of Jing Liu^37^. However, in contrast to the results of Sarah J^20^, the levels of β-catenin and Notch1 showed no significant increase in the ALDH+ population in our study^15^. Studies have shown that endostatin can target vascular endothelial cells through inhibition of Wnt signaling pathways and in this way display an anti-angiogenesis effect^40^,^41^,^42^.

In our study, we found that there was no significant difference neither in the viability of LLC cells nor in the percentage of ALDH+ cells between control and endostatin-cocultured LLC cells *in vitro*. However, *in vivo,* the percentage of ALDH+ cells from endostatin-treated mice was significantly greater than that from the control group. This result suggests that endostatin can increase the number of CSLCs by altering the tumor microenvironment.

There is much evidence showing that normal stem cells become located in hypoxic niches^51^. Additionally, at lower O_2_ tensions, hypoxia-inducible factor 1α (HIF-1α) facilitates signal transduction pathways, promotes the self-renewal, and prevents the differentiation of neural stem cells^49^. Moreover, Li Z. *et al*. reported that the effects of hypoxia are mainly mediated by hypoxia-inducible factors (HIFs), with HIF-2α being of particular importance for the GBM CSLC pool, increasing their self-renewal and tumorigenic capacity^51^. In the present study, treatment with endostatin was found to suppress formation of new vasculature, decrease levels of VEGF and increase HIF1-α expression in tumors, thus aggravating hypoxia in the tumor microenvironment. Furthermore, in a stimulated hypoxia condition, ALDH+ populations in the LLC increased. This observation indicated that endostatin increased CSLC levels by suppressing the formation of new vasculature and by aggravating hypoxia in the tumor.

There is now overwhelming evidence that the behavior of CSLCs is also highly influenced by their microenvironments^49^. In fact, the generation of inflammatory and immunosuppressive microenvironments play an important role in enriching CSLCs and maintain stem cell properties. Studies have shown TAMs^12^, MDSCs^13^ and Treg cells^14^ to be attracted into hypoxic areas, and these cells use paracrine signaling to produce many cytokines. which can aggravate the formation of inflammatory and immunosuppressive microenvironments. Additionally, TAMs, MDSCs, and Treg cells cross-talk with CSLCs to influence their stem cell properties through such production of cytokines. Irina Daurkin and colleagues reported that TAMs regulate murine breast cancer stem cells through a novel paracrine EGFR/Stat3/Sox-2 signaling pathway^50^. The action of MDSCs leads to an increased abundance of TGF-β in tumors, and induced EMT may be involved in the generation of CSLCs^52^,^53^. Gui TX and colleagues discovered that MDSCs enhance the “stemness” of cancer cells by inducing microRNA101 and suppressing the corepressor CtBP2^16^. Several recent reports have demonstrated that Treg cells, under certain circumstances, express IL-17,TGF-β, and VEGF, which together with hypoxia play a critical role in the regulation of cancer-initiating cells^18^,^54^,^55^,^56^. The present study revealed greater percentages of MDSCs, TAMs and Treg cells in tumors from endostatin-treated groups than from the controls. These cells were involved in resistance to endostatin. Take Treg cells for example. Endostatin treatment lead to increased number of Treg cells, which then resulted to higher level of cytokines, like TGF-β. As we discussed above that TGF-β was capable of inducing dedifferentiation of cancer cells into cancer stem-initiating cells through EMT. Moreover, the levels of EGF and IL-6 were increased in the treated groups.

The present study, however, also showed that anti-angiogenesis therapy can increase the numbers of CSLCs via generation of intratumor hypoxia and may involve the above cytokines and growth factors. Experiments have not yet been conducted on the intervention of each factor alone or in combination in mice. Carrying out related experiments would not only reveal the mechanisms by which antiangiogenesis therapy leads to an increase in the levels of CSLCs, but also may provide a new way to improve the clinical efficacy of antiangiogenic treatments.

In conclusion, endostatin treatment causes the levels of CSLCs in tumor microenvironments to increase, and both aggravated intratumoral hypoxia and attracted cytokine-secreting and growth factor-secreting TAMs, MDSCs and Treg cells in the tumor are related to the increased proportion of CSLCs. Our findings therefore imply that improving the clinical efficacy of antiangiogenic treatments will require the concurrent use of CSLC-targeting agents, which have significant clinical implications.

## Additional Information

**How to cite this article**: Yu, Y. *et al*. Antiangiogenic therapy using endostatin increases the number of ALDH+ lung cancer stem cells by generating intratumor hypoxia. *Sci. Rep.* **6**, 34239; doi: 10.1038/srep34239 (2016).

## Figures and Tables

**Table 1:** Primers used for RT- PCR analysis.

Gene		
Nanog	Primer forward 5′	5′ TGCTCCGCTCCATAACTTCG 3′
	Primer reverse 5′	5′ GCATGGCTTTCCCTAGTGGC 3′
Oct4	Primer forward	5′GCGGAGGGATGGCATACTGT 3′
	Primer reverse	5′TTCCCTCATCTCCAACTTCACG 3′
Sox2	Primer forward	5′ ACCAGCTCGCAGACCTACATG 3′
	Primer reverse	5′ GCCTCGGACTTGACCACAGA 3′
Shh	Primer forward	5′ GAAGATCACAAGAAACTCCGAACG3′
	Primer reverse	5′GCATTTAACTTGTCTTTGCACCTC 3′
Notch1	Primer forward	5′CAATCAGGGCACCTGTGAGCCCACAT3′
	Primer reverse	5′ TAGAGCGCTTGATTGGGTGCTTGCGC 3′^57^
β-Catenin	Primer forward	5′CCTTGGGACTCTAGTGCAGC 3′
	Primer reverse	5′ GTCGTGGAATAGCACCCTGT 3′^58^
GAPDH	Primer forward	5′ ACCTCAACTACATGGTCTAC 3′
	Primer reverse	5′ TTGTCATTGAGAGCAATGCC 3′^2^,^59^

## References

[b1] Folkman J. Tumor angiogenesis: Therapeutic implications. N Engl Med. 285, 1182–1186 (1971). 10.1056/NEJM197111182852108 4938153

[b2] Bergers G. & Hanahan D. Modes of resistance to anti-angiogenic therapy. Nat Rev Cancer. 8, 592–603 (2008). 18650835 10.1038/nrc2442 PMC2874834

[b3] Hayes D. F. Bevacizumab treatment for solid tumors: Boon or bust? JAMA. 305, 506–508 (2011). 21285431 10.1001/jama.2011.57

[b4] Burstein H. J. . Phase II study of sunitinib malate, an oral multitargeted tyrosine kinase inhibitor, in patients with metastatic breast cancer previously treated with an anthracycline and a taxane. J Clin Oncol. 26, 1810–1816 (2008). 18347007 10.1200/JCO.2007.14.5375

[b5] Johannsen M. . Can tyrosine kinase inhibitors be discontinued in patients with metastatic renal cell carcinoma and a complete response to treatment? A mul-ticentre, retrospective analysis. Eur Urol. 55, 1430–1438 (2009). 18950936 10.1016/j.eururo.2008.10.021

[b6] Shojaei F. Anti-angiogenesis therapy in cancer: current challenges and future perspectives [J]. Cancer Lett. 320, 130–137 (2012). 22425960 10.1016/j.canlet.2012.03.008

[b7] Polyak K. .Co-evolution of tumor cells and their microenvironment. Trends Genet. 25, 30–38 (2009). 19054589 10.1016/j.tig.2008.10.012

[b8] Lu X. & Kang Y. Hypoxia and hypoxia-inducible factors: Master regulators of metastasis. Clin Cancer Res. 16, 5928–5935 (2010). 20962028 10.1158/1078-0432.CCR-10-1360 PMC3005023

[b9] Soeda A. . Hypoxia promotes expansion of the CD133-positive glioma stem cells through activation of HIF-1 α. Oncogene. 28, 3949–3959 (2009). 19718046 10.1038/onc.2009.252

[b10] Seidel S., Garvalov B. K. . A hypoxic niche regulates glioblastoma stem cells through hypoxia inducible factor 2α. Brain. 133, 983–995 (2010). 20375133 10.1093/brain/awq042

[b11] Heddleston J. M. . The hypoxic microenvironment maintains glioblastoma stem cells and promotes reprogramming towards a cancer stem cell phenotype. Cell Cycle. 8, 3274–3284 (2009). 19770585 10.4161/cc.8.20.9701 PMC2825672

[b12] Conley S. J. . Antiangiogenic agents increase breast cancer stem cells via the generation of tumor hypoxia. Proc Natl Acad Sci USA 109, 2784–9 (2012). 22308314 10.1073/pnas.1018866109 PMC3286974

[b13] Doedens A. L. . Macrophage expression of hypoxia- inducible factor-1 alpha suppresses T-cell function and promotes tumor progression. Cancer Res. 70, 7465–7475 (2010). 20841473 10.1158/0008-5472.CAN-10-1439 PMC2948598

[b14] Corzo C. A. . HIF-1 regulates function and differentiation of myeloid-derived suppressor cells in the tumor microenvironment. J.Exp.Med. 207, 2439–2453 (2010). 20876310 10.1084/jem.20100587 PMC2964584

[b15] Clambey E. T. . Hypoxia-inducible factor-1 alpha-dependent induction of FoxP3 drives regulatory T-cell abundance and function during inflammatory hypoxia of the mucosa. Proc. Natl. Acad. Sci. USA. 109, 2784–2793 (2012). 22988108 10.1073/pnas.1202366109 PMC3478644

[b16] Daurkin I. . Tumor-Associated Macrophages Mediate Immunosuppression in the Renal Cancer Microenvironment by Activating the 15-Lipoxygenase-2 Pathway. Cancer Res. 71, 6400–9 (2011). 21900394 10.1158/0008-5472.CAN-11-1261

[b17] Cui T. X. . Myeloid-Derived Suppressor Cells Enhance Stemness of Cancer Cells by Inducing MicroRNA101 and Suppressing the C orepressor CtBP2. Immunity. 39, 611–21 (2013). 24012420 10.1016/j.immuni.2013.08.025 PMC3831370

[b18] Yu X. . Interaction between regulatory T cells and cancer stem cells. Int J Cancer J Int du Cancer. 131, 1491–8 (2012). 10.1002/ijc.27634 22592629

[b19] Sonja L. . Mechanisms of Resistance to Anti-Angiogenic Therapy and Development of Third-Generation Anti-Angiogenic Drug Candidates. Genes Cancer. 1, 12–25 (2010). 21779425 10.1177/1947601909356574 PMC3092176

[b20] Brahimi-Horn M. C., Chiche J. & Pouyssegur J. Hypoxia and cancer. J Mol Med. 85, 1301–7 (2007). 18026916 10.1007/s00109-007-0281-3

[b21] Dong Z. & Wang J. Hypoxia selection of death-resistant cells: a role for Bcl-X(L). J Biol Chem. 279, 9215–21 (2004). 14676192 10.1074/jbc.M312225200

[b22] Fang J. S., Gillies R. D. & Gatenby R. A. Adaptation to hypoxia and acidosis in carcinogenesis and tumor progression. Semin Cancer Biol. 18, 330–7 (2008). 18455429 10.1016/j.semcancer.2008.03.011 PMC2953714

[b23] Yu J. L. . Effect of p53 status on tumor response to antiangiogenic therapy. Science. 295, 1526–8 (2002). 11859195 10.1126/science.1068327

[b24] Keith B. & Simon M. C. Hypoxia-inducible factors, stem cells, and cancer. Cell. 129, 465–72 (2007). 17482542 10.1016/j.cell.2007.04.019 PMC3150586

[b25] Calabrese C. . A perivascular niche for brain tumor stem cells. Cancer Cell. 11, 69–82 (2007). 17222791 10.1016/j.ccr.2006.11.020

[b26] Zhang W. C. . Glycine decarboxylase activity drives non-small cell lung cancer tumor-initiating cells and tumorigenesis. Cell. 148, 259–272 (2012). 22225612 10.1016/j.cell.2011.11.050

[b27] Bertolini G. . Highly tumorigenic lung cancer CD133+ cells display stem-like features and are spared by cisplatin treatment. Proceedings of the National Academy of Sciences of the United States of America. 106, 16281–16286 (2009). 19805294 10.1073/pnas.0905653106 PMC2741477

[b28] Jung M. J. . Upregulation of CXCR4 is functionally crucial for maintenance of stemness in drug-resistant non-small cell lung cancer cells. Oncogene. 32, 209–221 (2013). 22370645 10.1038/onc.2012.37

[b29] Sullivan J. P. . Aldehyde dehydrogenase activity selects for lung adenocarcinoma stem cells dependent on notch signaling. Cancer research. 70, 9937–9948 (2010). 21118965 10.1158/0008-5472.CAN-10-0881 PMC3058307

[b30] Giang A. . Lung cancer and lung stem cells-strange bedfellows? Am J Resp and Criti Care Med. 175, 547–53 (2007). 10.1164/rccm.200607-984PP 17158280

[b31] Moreb J. . Human aldehyde dehydrogenase class I results in increased resis-tance to 4-hydropero xycyclophospha mide. Cancer Gene Ther. 3, 24–30 (1996). 8785707

[b32] Charafe J. E. . Aldehyde dehydrogenase 1-positive cancer stem cells mediate metastasis and poor clinical outcome in inflammatory breast cancer. Clin Cancer Res. 16, 45–55 (2010). 20028757 10.1158/1078-0432.CCR-09-1630 PMC2874875

[b33] Ueda K. . Aldehyde Dehydrogenase 1 Identifies Cells with Cancer Stem Cell-Like Properties in a Human Renal Cell Carcinoma Cell Line. PLoS ONE. 8, e75463 (2013). 24116047 10.1371/journal.pone.0075463 PMC3792959

[b34] Ping M. & Kaushal Joshi. Mesenchymal glioma stem cells are maintained by activated glycolytic metabolism involving aldehyde dehydrogenase 1A3. Proc Natl Acad Sci USA 110, 8644–9 (2013). 23650391 10.1073/pnas.1221478110 PMC3666732

[b35] Yu L., Nicholas N. & Mayumi F. Isolation of Human Melanoma Stem Cells Using ALDH as a Marker. Curr Protoc Stem Cell Biol. 26, Unit–3.8 (2013). 10.1002/9780470151808.sc0308s26 PMC4164114 24510792

[b36] Anitha S. & Elizabeth B. ALDH as a Marker for Enriching Tumorigenic Human Colonic Stem Cells. Methods Mol Biol. 916, 373–385 (2012). 22914954 10.1007/978-1-61779-980-8_27 PMC3975159

[b37] Liu J. . Lung cancer tumorigenicity and drug resistance are maintained through ALDH(hi)CD44(hi) tumor initiating cells. Oncotarget. 4, 1698–1711 (2013). 24091605 10.18632/oncotarget.1246 PMC3858556

[b38] Kumar P. A. . Distal airway stem cells yield alveoli *in vitro* and during lung regeneration following H1N1 influenza infection. Cell. 147, 525–538 (2011). 22036562 10.1016/j.cell.2011.10.001 PMC4040224

[b39] Jiang F. . Aldehyde dehydrogenase 1 is a tumor stem cell-associated marker in lung cancer. Mol Cancer Res. 7, 330–338 (2009). 19276181 10.1158/1541-7786.MCR-08-0393 PMC4255559

[b40] Ling Y. . Endostar, a novel recombinant human Endostatin, exerts antiangiogenic effect via blocking VEGF-induced tyrosine phosphorylation of KDR/Flk-1 of endothelial cells. Biochem Biophys Res Commun. 361, 79–84 (2007). 17644065 10.1016/j.bbrc.2007.06.155

[b41] Wang H. L. . Effect of Endostatin on preventing postoperative progression of distant metastasis in a murine lung cancer model. Tumori. 6, 787–793 (2011). 22322847 10.1177/030089161109700617

[b42] Folkman J. Antiangiogenesis in cancer therapy - Endostatin and its mechanisms of action. Exp Cell Res. 312, 594–607 (2006). 16376330 10.1016/j.yexcr.2005.11.015

[b43] Tao N. . Gene Therapy with the Angiogenesis Inhibitor Endostatin in an Orthotopic Lung Cancer Murine Model [J]. Human Gene Therapy. 20, 103–111 (2009). 18939902 10.1089/hum.2008.098

[b44] Ning T. . Low-dose Endostatin normalizes the structure and function of tumor vasculature and improves the delivery and anti-tumor efficacy of cytotoxic drugs in a lung cancer xenograft murine model. Thoracic Cancer. 3, 229–238 (2012). 10.1111/j.1759-7714.2012.00111.x 28920305

[b45] Hui Z. . Tumor refractoriness to Endostatin anti-angiogenesis is associated with the recruitment of CD11b+ Gr1+ myeloid cells and inflammatory cytokines. Tumori. 99, 708–718 (2013). 24503797 10.1177/030089161309900613

[b46] Charafe J. E. . Breast cancer cell lines contain functional cancer stem cells with metastatic capacity and a distinct molecular signature. Cancer Res. 69, 1302–1313 (2009). 19190339 10.1158/0008-5472.CAN-08-2741 PMC2819227

[b47] Oliver K. & Christian M. Continuous Administration of Endostatin by Intraperitoneally Implanted Osmotic Pump Improves the Efficacy and Potency of Therapy in a Mouse Xenograft Tumor Model. Cancer Res. 61, 7669–7674 (2001). 11606410

[b48] Singh . EGFR/Src/Akt signaling modulates Sox2 expression and self-renewal of stem-like side-population cells in non-small cell lung cancer. Molecular Cancer. 11, 73 (2012). 23009336 10.1186/1476-4598-11-73 PMC3497614

[b49] Clarke L. & van der K. D. Low Oxygen Enhances Primitive and Definitive Neural Stem Cell Colony Formation by Inhibiting Distinct Cell Death Pathways. Stem Cells. 27, 1879–86 (2009). 19544448 10.1002/stem.96 PMC2771103

[b50] Yang J. . Tumor-Associated Macrophages Regulate Murine Breast Cancer Stem Cells Through a Novel Paracrine EGFR/Stat3/Sox-2 Signaling Pathway. Stem Cells. 31, 248–58 (2013). 23169551 10.1002/stem.1281

[b51] Li Z. . Hypoxia-inducible factors regulate tumorigenic capacity of glioma stem cells. Cancer Cell. 15–50, 1–13 (2009). 10.1016/j.ccr.2009.03.018 PMC2693960 19477429

[b52] Prashant T. & William E. Carson, III. Signaling pathways involved in MDSC regulation. Biochim Biophys Acta. 1846, 55–65 (2014). 24727385 10.1016/j.bbcan.2014.04.003 PMC4140957

[b53] Yang L. . Abrogation of TGF beta signaling in mammary carcinomas recruits Gr-1+ CD11b+ myeloid cells that promote metastasis. Cancer Cell. 13, 23–35 (2008). 18167337 10.1016/j.ccr.2007.12.004 PMC2245859

[b54] Yang S. . Foxp3 þIL-17þ T cells promote development of cancer-initiating cells in colorect al cancer. J Leukoc Biol. 89, 85–91 (2011). 20952660 10.1189/jlb.0910506

[b55] Facciabene A. .Tumour hypoxia promote s tolerance and angiogenesis via CCL28 and Treg cells. Nature. 475, 226–30 (2011). 21753853 10.1038/nature10169

[b56] Gupta S. . Intratumoral FOXP3 expression in infiltrating breast carcinoma: its association with clinic opathologic parameter s and angiog enesis. Acta Onco. 46, 792–7 (2007). 10.1080/02841860701233443 17653902

[b57] Damia G. & D’Incalci M. Contemporar y pre-clinical develop-ment of anticancer agents-what are the optimal preclinical models? Eur J Cancer. 45, 2768–2781 (2009). 19762228 10.1016/j.ejca.2009.08.008

[b58] Folkman J. Angiogenesis: An organizing principle for drug discovery? Nat Rev Drug Discov. 6, 273–286 (2007). 17396134 10.1038/nrd2115

[b59] Choi D. . *In Vitro* Differentiation of Mouse Embryonic Stem Cells: Enrichment of Endodermal Cells in the Embryoid Body. Stem Cells. 23, 817–27 (2005). 15917477 10.1634/stemcells.2004-0262

